# Oncogenic secretory clusterin in hepatocellular carcinoma: Expression at early staging and emerging molecular target

**DOI:** 10.18632/oncotarget.13674

**Published:** 2016-11-29

**Authors:** Wenjie Zheng, Min Yao, Qi Qian, Wenli Sai, Liwei Qiu, Junling Yang, Wei Wu, Zhizhen Dong, Dengfu Yao

**Affiliations:** ^1^ Research Center of Clinical Medicine, Affiliated Hospital of Nantong University, Nantong 226001, Jiangsu Province, China; ^2^ Department of Immunology, Medical School of Nantong University, Nantong 226001, Jiangsu Province, China; ^3^ Department of Oncology, Yancheng 1^st^ People's Hospital, Yancheng 224005, Jiangsu Province, China; ^4^ Department of Diagnostics, Affiliated Hospital of Nantong University, Nantong 226001, Jiangsu Province, China

**Keywords:** hepatocellular carcinoma, secretory clusterin, prognosis, tumor stage, tumor growth

## Abstract

Secretory clusterin (sCLU) is associated with hepatocellular carcinoma (HCC) progression by contributing to angiogenesis, chemoresistance, cell survival, and metastasis. However, the sCLU expression at early stage of HCC progression remains to be clarified. In this study, the alteration of sCLU oncogenicity was firstly evaluated in HCC- and their para-cancerous- tissues. The incidence of sCLU expression in HCC was significantly higher than that in their non-tumorous tissues at message RNA (mRNA) or protein level, gradually increasing with tumor-node-metastasis (TNM) staging. Abnormal sCLU expression was associated with the poor differentiation, TNM stage, and considered as an independent prognostic factor for HCC patients. Furthermore, silencing sCLU gene transcription inhibited the colony formation and proliferation of HCC cells, with decreasing phosphorylation level of AKT and GSK-3β in HCCLM3 cells *in vitro* and significantly suppressed the HCC xenograft growth *in vivo*, suggesting that sCLU with oncogenicity should be not only an early indicator but also novel potential molecular-targeted therapy for HCC.

## INTRODUCTION

Hepatocellular carcinoma (HCC) is one of the most common malignant cancers and the 3^rd^ most frequent cause of cancer death worldwide [1–3]. Hepatitis B virus (HBV) or hepatitis C virus (HCV) infection along with alcohol and aflatoxin B1 intake are widely recognized as etiological agents in HCC [4–6]. Rapid progression, insensitivity to radiotherapy or chemotherapy, extensive metastasis and recurrence after surgery lead to a poor prognosis of HCC [7–10]. Therefore, improving the early diagnosis and searching for an effective treatment become urgent problems. To date, a few markers such as hepatoma-specific γ-glutamyl transferase, hepatoma-specific AFP, oncofetal antigen glypican-3, and member 3a of Wingless-type MMTV integration site family have been developed as specific biomarkers for HCC [11–13]. Given the unsatisfactory results for prognosis, however, it needs to explore novel markers and molecular-targets for HCC [14].

Clusterin (CLU) is a highly conserved heterodimeric disulfide-linked glyco- protein (originally named Apo-J), which is widely distributed in tissues and body fluids [15]. CLU is involved in various physiological processes, such as lipid transport, apoptosis, complements cascade, DNA repair, and cell adhesion [16–18]. The mature isoform of CLU is secretory CLU (sCLU) that mainly localizes in cytoplasm and over-expresses in a wide variety of tumors with oncogenicity [19,20]. Recently, abnormality of sCLU level was reported to correlate closely with HCC [21,22], such as sCLU-induced epithelial-mesenchymal transition [23], chemoresistance or metastasis [24,25], pro-hepatocarcinogenesis activity of sCLU *in vitro* models, interaction with oncogenes or suppressor genes, and cancer-associated pathways [26]. However, the mechanisms of sCLU in HCC progression or effects on HCC growth *in vivo* still remain to be clarified. Therefore, the current study was to analyze the alteration of hepatic sCLU in HCC at different staging, and inhibition of sCLU gene transcription by specific shRNA on effects of HCC growth *in vitro* and *in vivo*.

## RESULTS

### Hepatic sCLU expression and TNM staging of HCC

**Figure 1:**
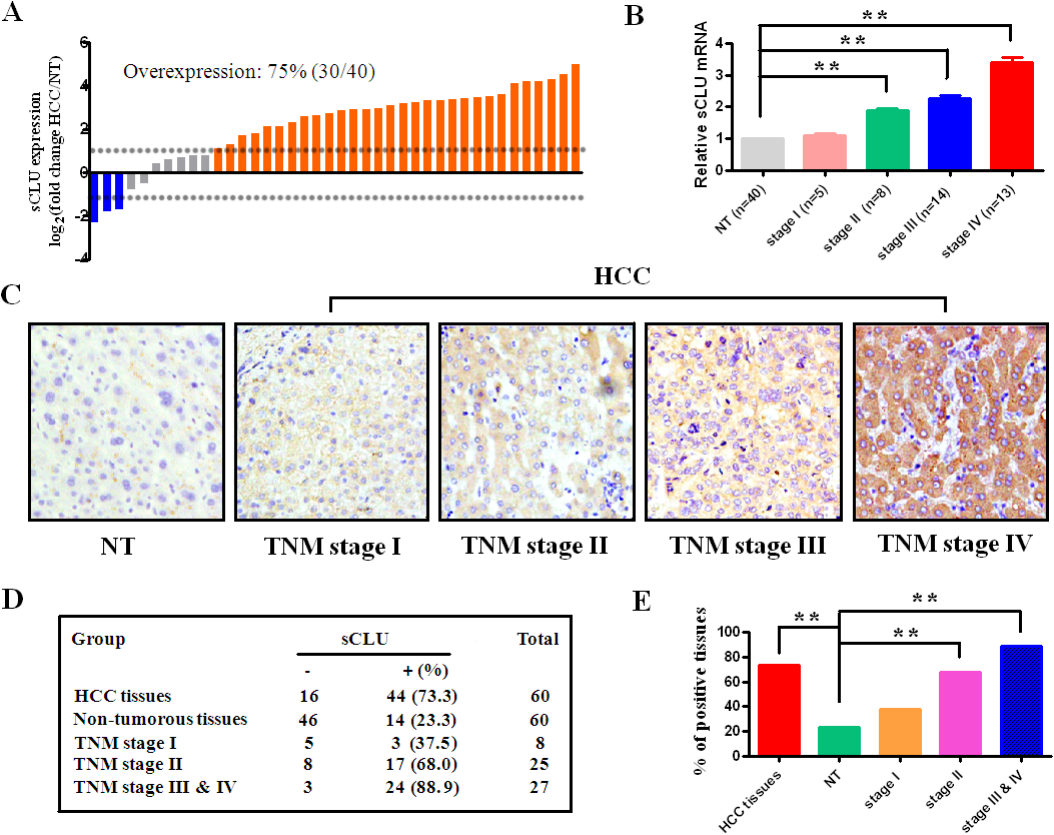
sCLU expression inHCC tissues **A**. sCLU mRNA expression in 40 pairs of fresh HCC tissues and nontumorous tissues (NT) detected by qRT-PCR. GAPDH was used as an internal control. The dotted line represented the fold change of sCLU equal to 2. **B**. sCLU mRNA expression in NT and HCC with different TNM stages determined by qRT-PCR. **C**. the representative sCLU immunohistochemical staining of the HCC or corresponding NT in the tissue microarrays (Original magnification × 400); **D**. the case numbers and percentages according to the sCLU expression and TNM stages of HCC; **E**. the bar graph summary of data presented in Figure 1D. **HCC**, hepatocellular carcinoma; **NT**, nontumorous tissues; **sCLU**, secretory clusterin; **TNM**, tumor-node-metastasis. **, *P*< 0.01.

Hepatic sCLU expression in 40 pairs of fresh HCC- and their non-tumorous- tissues (NT) is shown in Figure 1. The sCLU expression at messenger RNA (mRNA) level was observed in cancerous or non-tumorous tissues by quantitative real-time polymerase chain reaction (qRT-PCR, Figure 1A). The overall level of sCLU mRNA[log_2_(HCC/NT)>1] in HCC was 75% up-regulated (30/40), 7.5% down-regulated (3/40), and 17.5% non-changed (7/40). No significant difference at staging I was found between NT and HCC. However, the sCLU mRNA level was drastically up-regulated from staging II to IV (Figure 1B). The sCLU expressions at protein level in 60 HCC and their NT tissues were analyzed by the tissue microarray (TMA) with immunohistochemistry. As presented in Figure 1C, the sCLU staining was mainly presented in the cytoplasm. The positive rate of sCLU expression in the HCC tissues (73.3%, 44/60) was significantly higher (*χ^2^*=30.033, *P*<0.001) than that in the NT group (23.3%, 14/60, Figure 1D). Moreover, the incidence of sCLU expression in HCC was 37.5 % (3/8) at staging I, 68% (17/25) at staging II, and 88.9% (24/37) at staging III & IV, respectively (Figure 1E). The levels of sCLU protein consistent with their mRNA expression were gradually up-regulation with increasing HCC staging.

### Clinicopathological features of high sCLU expression

**Figure 2:**
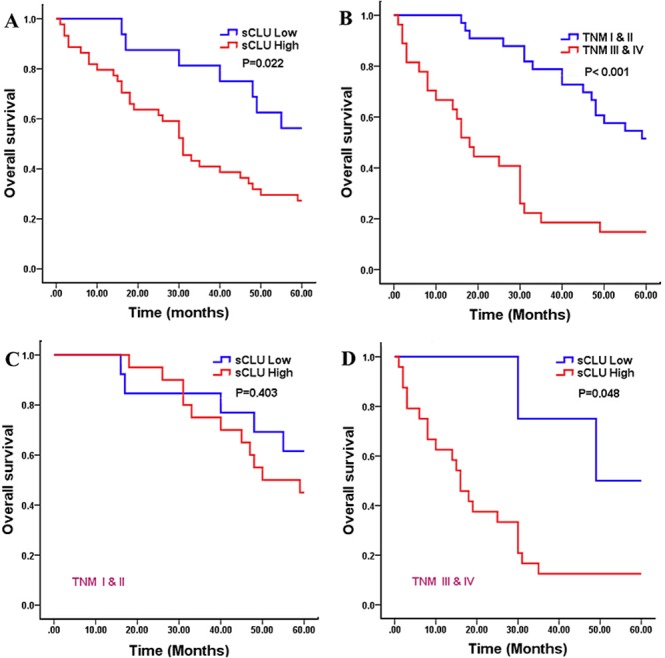
Kaplan-Meier analysis for overall survival of 60 HCC patients The survival curves were calculated according to the sCLU expression or TNM stages of HCC. Two-sided log-rank tests were performed to evaluate the statistical significance. **A**. the 5-year overall survival curve made according to high or low sCLU expression (*P*=0.022); **B**. the survival curve made according to high or low TNM stages (*P*<0.001); **C**. the 5-year overall survival curve made according to sCLU expression in HCC patients at I ∼ II stages (*P*=0.403); **D**. the 5-year overall survival curve made according to sCLU expression in HCC at III ∼ IV stages (*P*=0.048). The red line is the higher sCLU group; and the blue line is the lower or negative sCLU group. **HCC**, hepatocellular carcinoma; **sCLU**, secretory clusterin; **TNM**, tumor-node-metastasis.

The associations between sCLU and clinicopathological parameters in TMA containing 60 HCC cases are elucidated in Table 1. Results revealed that high sCLU expression was significantly linked to poor differentiation (*χ^2^*=4.651, *P*=0.031) and advanced TNM stage (*χ^2^*=6.074, *P*=0.014). However, no significant associations were found between sCLU expression and patients’ age, gender, AFP level, portal vein invasion, HBV infection, tumor size, liver cirrhosis, lymph node metastasis, and gross classification. Furthermore, the Kaplan-Meier survival curves with log-rank tests were performed to evaluate the prognostic value of sCLU (Figure 2). Compared with low expression of sCLU, there was a trend toward a poorer overall survival in HCC patients with positive sCLU expression (*χ^2^*=5.208, *P*=0.022, Figure 2A). Besides, survival time of HCC patients with high TNM stage was significantly shorter than that of cases with low stage (*χ^2^*=19.518, *P*<0.001, Figure 2B). Moreover, in the subset of HCC patients with TNM III & IV stage, high sCLU expression was prone to result in a shorter survival time compared with low sCLU expression (*χ^2^*=3.920, *P*=0.048, Figure 2D); however, no obvious difference of survival time was found in patients at staging I & II according to high or low sCLU expression (*χ^2^*=0.700, *P*=0.403, Figure 2C).

**Table 1:** Correlations of sCLU expression with clinical factors in HCC

Parameters	n	sCLU positive n (%)	*χ^2^* value	*P* value
Age (years)			0.135	0.713
<50	21	16(76.2)		
≥50	39	28(71.8)		
Gender			2.532	0.112
Male	39	26(66.7)		
Female	21	18(85.7)		
AFP(μg /L)			0.156	0.693
<50	25	19(76.0)		
≥50	35	25(71.4)		
Portal vein invasion			0.002	0.967
With	19	14(73.7)		
Without	41	30(73.2)		
HBsAg			2.431	0.119
Positive	49	38(77.6)		
Negative	11	6(54.6)		
Tumor size			0.024	0.876
<5 cm	31	23(74.2)		
≥5 cm	29	21(72.4)		
Liver cirrhosis			0.006	0.936
With	37	27(73.0)		
Without	23	17(73.9)		
Lymph node metastasis			2.578	0.108
With	12	11(91.7)		
Without	48	33(68.8)		
Gross classification			1.091	0.296
Multifocal	10	6 (60.0)		
Unifocal	50	38 (76.0)		
Differentiation			4.651	**0.031**
Well & Moderate	44	29(65.9)		
Poor	16	15(93.8)		
TNM			6.074	**0.014**
I & II	33	20(60.6)		
III & IV	27	24(88.9)		

Abbreviations: **AFP**, α-fetoprotein; **TNM**, tumor-node-metastasis; **sCLU**, secretory clusterin. Boldface, *P*<0.05.

### Univariate and multivariate analysis of sCLU expression

The univariate or multivariate Cox Regression analysis identifying factors associated with 5-year survival is listed in Table 2. The univariate Cox regression analysis demonstrated that sCLU expression, gross classification, lymph node metastasis, and TNM stage were significant prognostic factors influencing survival of HCC patients. And then, the multivariate Cox regression analysis was conducted to analyze the factors above, showed the sCLU expression associated with an increased risk of death, and recommended as an independent prognostic factor for the subset of HCC patients (hazard ratio, 2.684; 95% confidence interval: 1.072-6.722; *P*=0.035), along with the TNM stage (hazard ratio, 2.513; 95% confidence interval: 1.115-5.665; *P*=0.026).

**Table 2:** Univariate and multivariate analysis of sCLU expression in HCC tissues

	Univariate analysis			Multivariable analysis (adjusted for age and sex)		
	HR	*P*-value	95% CI	HR	*P*-value	95% CI
AFP (ng/ml)						
<50 *vs*. ≥50	1.293	0.423	0.690-2.425			
Portal vein invasion						
Yes *vs*. No	1.493	0.224	0.783-2.805			
HBsAg						
Yes *vs*. No	1.837	0.205	0.717-4.708			
Tumor size						
<5 cm *vs*. ≥5 cm	1.404	0.291	0.747-2.637			
Liver cirrhosis						
Yes *vs*. No	0.759	0.392	0.404-1.427			
Lymph node metastasis						
Yes *vs*. No	4.248	**<0.001**	2.051-8.795	2.411	0.063	0.953-6.102
Gross classification						
Multifocal *vs*. Unifocal	2.130	**0.049**	1.004-4.520	1.731	0.218	0.723-4.149
Differentiation						
Well, Moderate *vs*. Poor	1.131	0.729	0.563-2.274			
TNM						
I &II *vs*. III&IV	3.917	**<0.001**	2.036-7.537	2.513	**0.026**	1.115-5.665
sCLU expression						
Positive *vs*. Negative	2.494	**0.029**	1.097-5.670	2.684	**0.035**	1.072-6.722

Abbreviations: **CI**, confidence interval; **HR**, hazard ratio; **AFP**, α-fetoprotein; **TNM**, tumor-node-metastasis; **sCLU**, secretory clusterin. Boldface, *P*<0.05.

### Differences of sCLU expressions among HCC cell lines

**Figure 3:**
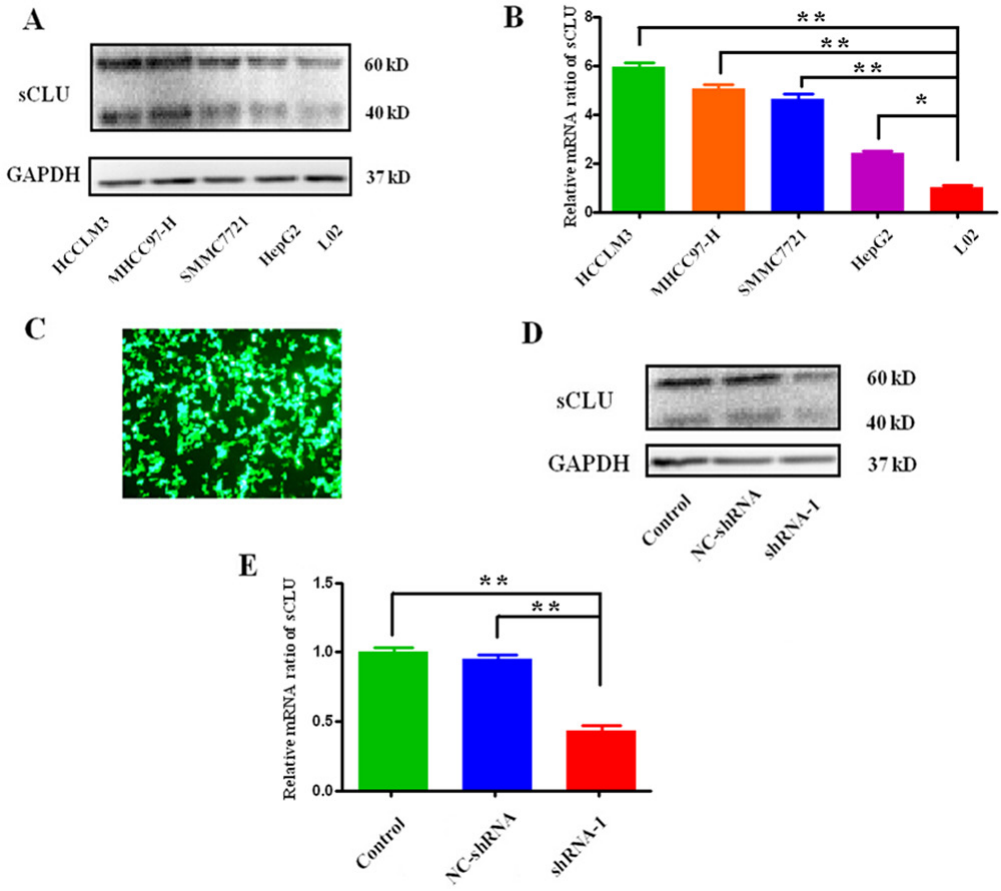
Expression of sCLU in HCC cells or silencing by specific shRNA Expression of sCLU in HCCLM3, MHCC97-H, SMMC7721, HepG2, and L02 cells was detected by Western blotting at protein level or qRT-PCR at mRNA level. Then, the HCCLM3 cells were transfected with the specific shRNA to abrogate sCLU expression. **A**. sCLU expression among the HCC cells analyzed by the Western blotting with GAPDH as the internal control; **B**. sCLU mRNA expression among the HCC cells analyzed by the qRT-PCR; **C**. the fluorescence photomicrographs after the HCCLM3 cells transfected with specific shRNA-1; **D**. the comparative analysis of sCLU expression in the group with or without shRNA-1 transfection detected by the Western blotting; **E**. the comparative analysis of sCLU mRNA expression in the group with or without shRNA-1 transfection detected by qRT-PCR. The data were presented as means ± SD (n=3). *, *P*<0.05; **, *P*<0.01.

The expression of sCLU among different human HCC cell lines is shown in Figure 3. As shown in Figure 3A and 3B, the expression of sCLU at protein or mRNA level in HCC cells (HCCLM3, MHCC97-H, SMMC-7721 and HepG2) was significantly higher than that in hepatocyte L02 cells. Furthermore, the effective shRNA-1 and a negative-control shRNA (NC-shRNA) were transfected into HCCLM3 cells, and the fluorescence photomicrographs were observed under a microscope (Figure 3C). As presented in Figure 3E, shRNA-1 could significantly decrease the expression of sCLU-mRNA in HCCLM3 (56.67%, *q*=18.19, *P*<0.001). Consistently, the sCLU protein expression was also obviously repressed in cells transfected with shRNA-1, in contrast to the control or NC-shRNA group (Figure 3D).

### Specific shRNA suppressed proliferation of HCC cells

**Figure 4:**
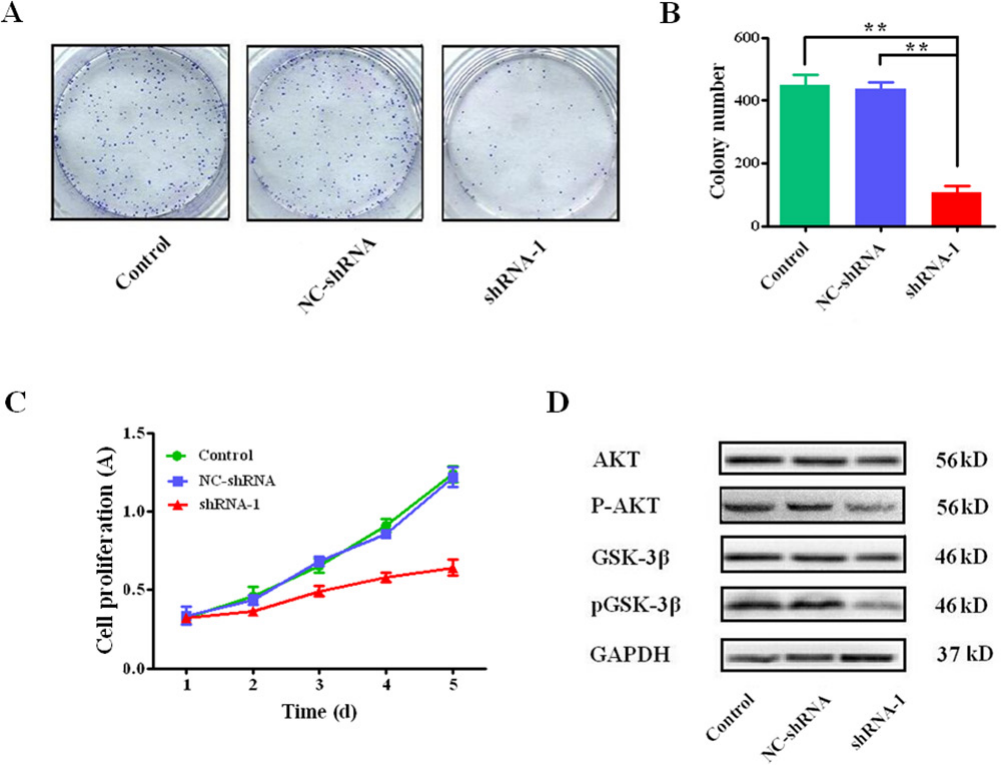
Silencing sCLU on effects of colony formation and cell proliferation **A**. the colony formation assay of HCC cells, and the representative micrographs from the Giemsa-stained colonies formed by HCCLM3 cells with transfection of shRNA-1, NC-shRNA or without transfection; **B**. the comparative analysis of the micrograph quantification results from the Giemsa-stained HCCLM3 cells transfected with shRNA-1, NC-shRNA or control group (n=3); **C**. the viability of HCCLM3 cells transfected with the shRNA-1, NC-shRNA or without transfection determined by CCK-8 assay on days 1 to 5; **D**. the levels of AKT, phosphorylated-AKT, GSK-3β, and phosphorylated-GSK-3β expressions detected by Western blotting. The data were presented as means ± SD (n=3). **, *P*< 0.01.

The colony formation and proliferation in HCC cells mediated by sCLU is presented in Figure 4. The colony numbers in the shRNA-1 group (105.3±22.8) were significantly less than these in the control group (447.3±34.2, *q*=21.88, *P*<0.001) or the NC-shRNA group (435.3±22.5, *q*= 21.12, *P*<0.001, Figure 4A), respectively. Besides, the CCK-8 assay showed that the proliferation of HCCLM3 cells was obviously repressed after inhibition of sCLU gene transcription by specific shRNA-1(Figure 4B). Furthermore, the protein levels of AKT and GSK-3β, which were crucial to HCC proliferation, were detected after sCLU silencing. The total protein levels of AKT and GSK-3β had no obvious changes, while p-AKT and p-GSK-3β expressions decreased significantly after knock-down of sCLU (Figure 4C).

### Silencing sCLU suppressed HCC growth *in vivo*

**Figure 5:**
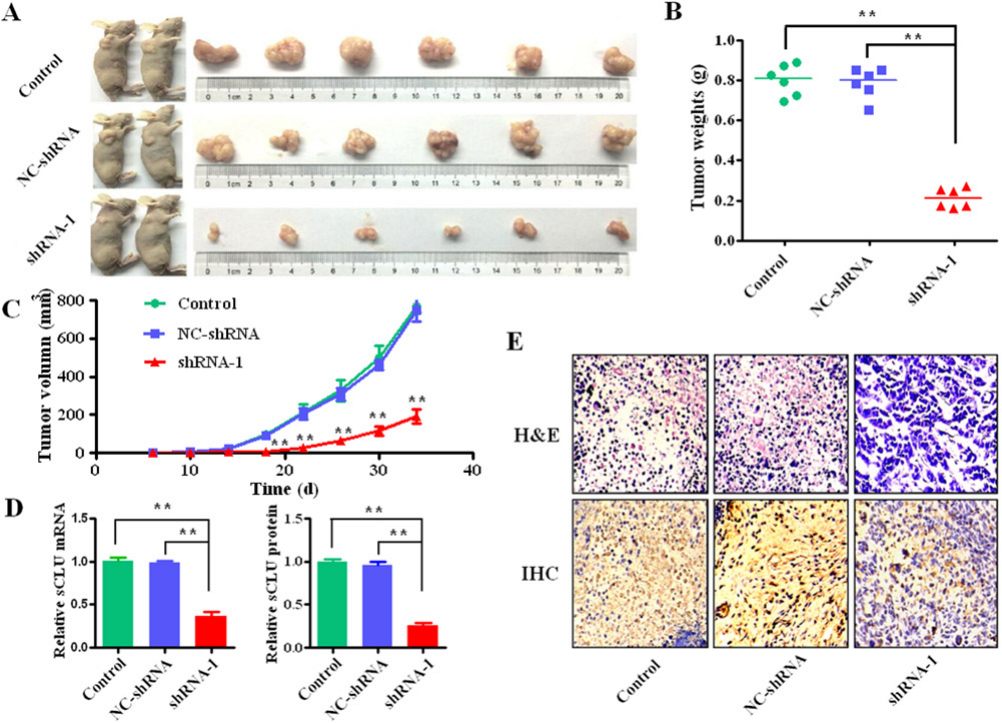
Intervening sCLU gene transcription on effect of xenograft growth HCCLM3 cells transfected with shRNA-1, NC-shRNA or without transfection were injected into nude mice to observe the formation of tumor. **A**. the representative photographs of the nude mice and corresponding dissected tumors from each group; **B**. the mean tumor weights in nude mice subcutaneously inoculated with the HCCLM3 cells transfected with shRNA-1 and NC-shRNA or without transfection as control; **C**. the growth curves of the xenograft tumors among the different groups, and the tumor volumes were measured every 4 days; **D**. the relative expression of sCLU mRNA in each group was detected by the qRT-PCR with comparative cycle threshold (Ct) method. The relative sCLU expression at protein level was calculated according to the ratio from tissue sCLU concentration to total protein by ELISA analysis; **E**. the patho-histology or the alteration of sCLU expression in the resected tumor tissues were examined by the H&E or immunohistochemical staining, respectively (Original magnification×400). The data were presented as means ± SD of duplicate experiments. **H&E**, Hematoxylin and Eosin; **IHC**, immunohistochemistry. **, *P*<0.01.

The tumor growth and morphology are shown in Figure 5. The mice were sacrificed at the 34^th^ day after injection (Figure 5A). The mean weight of the xenograft tumors in the shRNA-1 group (0.21±0.05 g) was significantly less than that of control group (0.8±0.07g, *q*=20.73, *P*<0.001) and the NC-shRNA group (0.78±0.08g, *q*=20.22, *P*<0.001; Figure 5B), respectively. Besides, growth curves of subcutaneously xenograft tumors indicated that the tumor growth of shRNA-1 group was significantly slower than that of the control and the NC-shRNA group after the 18^th^ day (Figure 5C). The sCLU mRNA level in the shRNA-1 group was significantly lower than that in the control (*q*=20.22, *P*<0.001) or the NC-shRNA group (*q*=20.22, *P*<0.001; Figure 5D). Consistently, the sCLU protein expression in the shRNA-1 group was also lower than that in the control (*q*=36.44, *P*<0.001) or the NC-shRNA group (*q*=35.58, *P*<0.001). In addition, the tumor morphological alterations of audio-videoailable atypia among the different groups were confirmed by hematoxylin-eosin staining (H&E). Moreover, the sCLU staining in the control or NC-shRNA group was stronger than that in the shRNA-1 group by immunohistochemistry (Figure 5E).

## DISCUSSION

HCC is one of the most frequently diagnosed cancers in the world. Mounting reports note that HCC development is a multi-step and multi-centric process with the activation of oncogenes and inactivation of tumor supressor genes [27]. Therefore, it is of great significance to find novel and effective targets against HCC. Recent studies indicate that sCLU is over-expressed in various aggressive neoplasms and plays crucial roles in the development and progression of malignancies [28]. In the present study, the oncogenic sCLU expression at different staging of HCC progression and its application value as a molecular target for HCC was investigated *in vitro* and *in vivo*.

Though previous studies determined the abnormal expression of sCLU and its prognostic value in HCC, the alteration of sCLU expression in different staging of HCC had not been explored [29]. The current data demonstrated that sCLU was over-expressed in HCC tissues at mRNA or protein level, which was significantly higher than that in the corresponding nontumorous tissues. Notably, our study found that sCLU mRNA and protein expression presented a gradual upregulation in the HCC cases from low clinical stage to high stage. Besides, hepatic sCLU increased drastically from the group of stage I to II, indicating a positive role in early progression of HCC. Moreover, high sCLU expression was significantly associated with poor differentiation, advanced TNM stage, and short survival time of HCC patients, suggesting that sCLU might promote the early progression of HCC and should be an early molecular biomarker for HCC.

Increasing evidence note that sCLU overexpression may promote proliferation of cancer cells [30, 31]. This study further conducted related assays on HCCLM3 cells to explore the potential functions of sCLU. Obvious inhibition of colony formation and proliferation was observed in HCCLM3 cells after knock-down of sCLU. Certain signal pathways might be involved in sCLU-mediated proliferation. Activation of AKT could deteriorate the activity of GSK-3β, subsequently up-regulating the oncogenes of the downstream, and ultimately promoting the proliferation of malignant cells [32–34]. Notably, our study discovered that silencing sCLU decreased phosphorylated AKT expression (active form) and phosphorylated GSK-3β expression (inactive form), indicating that AKT inactivation and GSK-3β activation might relate to proliferation of HCC cells.

Mounting studies demonstrate that sCLU is involved in the development of various malignancies. Abrogating sCLU expression could significantly inhibit the growth of tumor derived from cancer cells [35,36]. Conversely, sCLU-overexpression resulted in a fast growth in the orthotopic primary tumor [37]. Then, our study further explored the possible hepatocarcinogenesis function of sCLU *in vivo*. Obviously, the xenograft tumors formed by sCLU-silenced HCC cells grew much slower than the control group. Besides, it was validated that the sCLU expression in small xenograft tumor was significantly lower than that in large size tumor. Given the *in vivo* findings, sCLU may be considered to participate in the tumorigenicity of HCC.

In conclusion, the up-regulation of sCLU expression at early staging of HCC is considered to promote tumor development and exacerbate the survival of HCC patients, which may be related to the phosphorylation of AKT/GSK-3β. Targeted therapy offers an effective option for non-surgical HCC management, but it remains to be a challenge due to lack of specific targets. Silencing sCLU provides a new mechanism insight into molecular-targeted therapy for its inhibition of HCC growth. Admittedly, to clarify the exact role of sCLU more roundly and convincingly, larger number of clinical cases and delicate experiments could be adopted in further studies. It is expected that inhibiting sCLU gene transcription plus multi-targeting strategies should be explored for HCC therapy [38].

## MATERIALS AND METHODS

### Ethics statement

The study protocol was approved by the Research Ethics Committee of Affiliated Hospital of Nantong University (approval number: TDFY2012-1) and performed according to the World Medical Association Declaration of Helsinki [39]. Written or verbal consent was required from each patient involved in the study.

### Liver tissues

A total of 60 primary HCC- and their self-matched NT tissues were collected from HCC patients who received hepatectomy at the Affiliated Hospital of Nantong University, Nantong, China from Jan 2008 to Jan 2010. The diagnosis of HCC was confirmed histologically in each patient, and no patient underwent chemotherapy or radiotherapy before the surgery. Clinical information was obtained from medical records and included a 5-year post-surgery follow-up period. For each case, patient's gender, age, TNM stage, tumor size, differentiation, HBsAg infection, portal vein invasion, and lymph node metastasis status were recorded. In addition, 40 pairs of fresh HCC tissues and matched nontumorous tissues were obtained from the patients who underwent hepatectomy at the Affiliated Hospital of Nantong University from Sept 2012 to Sept 2014. After surgery, the liver tissues were immediately frozen at −80°C for further RNA isolation. The diagnosis of HCC, cirrhosis, and viral hepatitis was based on the criteria proposed by the Ministry of Health of the People's Republic of China and the Chinese National Viral Hepatitis Meeting, respectively.

### Tissue microarray (TMA) and immunohistochemistry (IHC)

TMA chip containing 60 cancerous- and matched NT- tissues were constructed by the Outdo Biotech Company (Shanghai, China). IHC assay was performed by using the Autostainer Universal Staining System (LabVision, USA) as a previous study [40]. In brief, the TMA sections were deparaffinized in xylene and quenched by 0.3% (V/V) hydrogen peroxide in methanol. After treating with citrate buffer and endogenous peroxidase, the slides were blocked in 10% goat serum to avoid nonspecific staining. And then slides were incubated overnight with mouse anti-human sCLU monoclonal antibody (1:200, Santa Cruz, USA) at 4°C. Following washing in phosphate buffered saline (PBS) for 3 times, the slides were incubated with horse reddish peroxidase-conjugated goat anti-mouse antibody (1:1000, DAKO, USA) at room temperature for 30min. Finally, the TMA sections were visualized with diaminobenzine and counter-stained with hematoxylin (Kem-En-Tec Diagnostics, Denmark).

### Evaluation of sCLU expression in HCC tissues

The IHC staining results of sCLU expression in TMA section were evaluated independently by two experienced pathologists using a semi-quantitative score system. IHC score of each sample was defined as the percentage sum of positive staining level (0, 1, 2, and 3) and the staining intensity (0, 1, 2, and 3). According to the IHC score, the cancerous tissues were divided into two groups: the low sCLU expression with 0∼2 scores and high expression with 3∼6 scores. Given the differences between duplicate tissue scores, the higher score was taken as the final score.

### Specific shRNA plasmid construction

The shRNAs targeting different sites of human sCLU gene according to the GenBank ID 001831.3 were designed and synthesized by Biomics Company (Nantong, China). And the most effective shRNA silencing sCLU was screened as the previously described [25]. NC-shRNA or effective shRNA-1 was constructed into pGPU6/GFP/Neo vectors, and inserted sequences were verified by sequencing. Sequences: shRNA-1, 5′-GTAAGTACGTCAATAAGGA-3′; and NC-shRNA, 5′-TTCTCCGAACGTGTCACGT-3′.

### Cell culture and transfection

Human hepatoma HCCLM3, SMMC7721, MHCC97-H, HepG2, and hepatocyte L02 cells were purchased from Chinese Academy of Sciences (Shanghai, China) and cultured in Dulbecco's modified Eagle's medium (DMEM, KeyGen Biotech, Nanjing, China) with 10% fetal bovine serum (FBS, Gibco, USA) at 37°C in a humidified incubator containing 5% CO_2_. Specific plasmids were transfected into cells with GenJet^TM^ DNA *in Vitro* Transfection Reagent (SignaGen, USA) according to the manufacturer's instructions. Briefly, cells were planted in 6-well plates at 24h prior to transfection. Fresh culture medium was replaced in each well at 1h before transfection. Then, 200 μL of DMEM mixture containing DNA and GenJet^TM^ (1:3) was cautiously added into each well. After incubating for 12h, the medium was replaced by fresh complete medium. Finally, the transfection efficiency was observed by the fluorescence microscope.

### Colony formation analysis

Human hepatoma cells (1×10^3^ cells/well) transfected with NC-shRNA, shRNA-1 or without transfection were seeded in 6-well plates and continuously incubated for 10 days. During the indicated time, the culture medium was replaced every 3 days. Then, cells were washed by phosphate buffer saline (PBS), fixed by methanol for 30 min, stained with Giemsa (Beyotime, Jiangsu, China). The single colony with more than 50 cells was counted under a stereomicroscope. The experiment was conducted in triplicate.

### Proliferation assay

Cell proliferation assay was detected by the Cell Counting Kit 8 (CCK-8, Beyotime, Jiangsu, China) according to the manufacturer's instructions. In brief, human hepatoma cells were plated in 96-well plates at a density of 5×10^3^ cells per well. After 24 h, the cells were transfected with NC-shRNA, shRNA-1 or without transfection. At the followed post-transfection time (1, 2, 3, 4, or 5 days), the cells was incubated in medium containing CCK-8 reagent for 4 h. After that, the absorbance at 450nm was measured by using a microplate reader. The assays were performed in triplicate.

### RNA isolation and qRT-PCR

Total RNA was isolated from tissues and cells with Trizol reagent (Invitrogen, USA) according to the manufacturer's instructions. Equal amounts of RNA were reverse transcribed into cDNA using the RevertAid^TM^ First Strand cDNA Synthesis Kit (MBI Fermentas, CA). qRT-PCR was conducted by SYBR®Premix Ex TaqTMII (TaKaRa, Dalian, China) with the manufacturer's instructions. Glyceraldehyde-3-phosphate dehydrogenase (GAPDH) was used as an internal control, and the melting curves were detected for the amplification specificity. The messenger RNA (mRNA) value was calculated based on the Ct values using the 2^−ΔΔCt^ method [ΔCt = Ct_target gene_ - Ct_GAPDH_]. The primers were as follows: sCLU, F: 3′-ATCACTGTGACGGTCCCTGTA-5′, and R: 3′-TCACTCCTCCCGGTGCTT-5′; GAPDH, F: 3′-CAAGGTCATCCATGACAACTTTG-5′, and R: 3′-GTCCACCACCCTGTTGCTGTAG-5′.

### Western blotting

Protein samples collected by sodium dodecyl sulfate (SDS) cell lysis buffer (Beyotime, Nanjing, China) were separated by SDS-polyacrylamide gel electro- phoresis for 1.5 h, and then transferred onto polyvinylidene fluoride membranes after 2 h. Following blocking for 2.5 h, the membranes were incubated in the primary antibody diluents overnight. Moreover, after washing with tris-buffered saline with tween (TBST), membranes were incubated in the secondary antibody diluents for 2 h. Finally, the samples were detected by electrochemiluminescence (ECL) kit (Millipore, USA). Antibodies were diluted as follows: GAPDH, AKT and GSK-3β (1:1000; Cell Signaling, USA); sCLU (1:500; Santa Cruz, USA); phos-AKT and phos-GSK-3β (1:1000; Abcam, USA); IgG horseradish peroxidase conjugate (1:1000; Univ-bio, Nanjing, China).

### Xenograft tumor models

All procedures on mice were performed according to the Guidelines for the Experimental Animals and approved by the Animal Care and Use Committee of Nantong University. The 6 weeks old BALB/c nude mice (n= 18) were provided by the Experimental Animal Center of Nantong University, and randomly assigned into three groups (sCLU-shRNA, NC-shRNA, and control). Briefly, hepatoma cells (2 × 10^7^ cells) that were stably transfected with sCLU-shRNA, NC-shRNA or without transfection were suspended in 200μL DMEM and injected subcutaneously into the right scapular area of nude mice. Tumor size was measured using calipers every 4 days. The tumor volume was calculated as follows: the tumor volume (mm^3^) = 0.5 (length×width^2^). The animals were sacrificed at the end of 5 weeks after injection. The xenograft tumors were dissected and fixed in 4% paraformaldehyde, embedded in paraffin, deparaffinized in xylene, and dehydrated in a gradient of ethanol solutions. Finally, histopathological examination was performed with the Hematoxylin & Eosin (H&E) staining.

### Protein extraction from xenograft tumors

Total proteins were extracted from the xenograft tumors of different groups by ice-cold radio immunoprecipitation assay lysis buffer (Beyotime, Shanghai, China). After centrifuged at 12,000 rpm for 30 min, the lysate supernatants were harvested. The total protein levels were measured by the Bicinchonininc Acid Protein Assay Kit (Beyotime, Shanghai, China) according to the manufacturer's instructions.

### ELISA analysis

Furthermore, protein level of sCLU was detected by the sCLU Enzyme-Linked Immunosorbent Assay (ELISA) Kit (Boster, Wuhan, China). In brief, 100μL lysate of each group was added into indicated wells and incubated at 37°C for 2 h. Then 100μL of the sCLU antibody solution and the avidin-biotin complex solution was added in sequence. Contents of wells were aspirated and washed 5 times. After that, 100μL of the tetramethyl benzidine development solution and 90μL of Stop solution were added to appropriate wells to stop reaction. Finally, the values at A_450_nm of duplicate samples were detected with a microplate reader (MD, USA).

### Statistical methods

Data were presented as means ± standard deviation (SD), and analyzed by using the SPSS19.0 Software. The relationships between clinical features and sCLU expression were determined by the χ^2^ test. Kaplan-Meier analysis with a log-rank test was performed to calculate the survival curves. The univariate and multivariate Cox regression analyses were conducted to evaluate the prognostic value. Comparisons among the Control, NC-shRNA, and shRNA-1 groups were performed by Student's Newman-Keuls (SNK) comparison. *P*<0.05 was considered to be statistically significant.

## Abbreviations

AFP: alpha-fetoprotein
ELISA: enzyme-linked immunosorbent assay
HCC: hepatocellular carcinoma
H&E: Hematoxylin and Eosin
IHC: immunohistochemistry
PBS: phosphate buffer saline
sCLU: secretory clusterin
TMA: tissue microarray
